# Case report: Systemic air embolism after percutaneous microwave ablation of an unresectable metastatic lung lesion

**DOI:** 10.3389/fonc.2024.1476346

**Published:** 2024-11-26

**Authors:** Wei Tang, Fandong Zhu, Jianfeng Yang, Ting Wang, Zhenhua Zhao, Jiangfeng Feng

**Affiliations:** ^1^ Department of Radiology, Shaoxing People’s Hospital, Shaoxing, China; ^2^ Department of Radiology, Shaoxing City Women and Children Hospital, Shaoxing, Zhejiang, China

**Keywords:** systemic air embolism, microwave ablation, metastatic lung lesions, case report, lung biopsy

## Abstract

Systemic air embolism, though rare, poses a significant risk as a complication of CT-guided lung lesion ablation. This condition often arises from the formation of a transient bronchovascular fistula, which can occur when a needle punctures the lung or accidentally breaches a pulmonary vein. In this report, we present a case involving acute cerebral infarction resulting from retrograde venous embolism linked to a fistula between the pleural cavity and pulmonary artery after pulmonary ablation. The patient demonstrated clear signs and symptoms but ultimately recovered fully and was discharged without lasting effects. This case underscores the critical need for ongoing vigilance and proactive measures to prevent air embolism during such medical procedures.

## Introduction

Percutaneous CT-guided microwave ablation (MWA) is a well-established and effective therapeutic approach for treating malignant lung tumors measuring up to 4 cm in diameter (1). Generally, MWA for lung tumors is considered a safe procedure, with predominantly minor complications; the most common of these is pneumothorax (2). Although air embolism is a rare complication following pulmonary ablation, it has been sporadically reported in the literature (3–5). This case report describes a patient who developed an air embolism after undergoing MWA, which was further complicated by a rapidly progressing stroke.

## Case report

**Figure 1:**
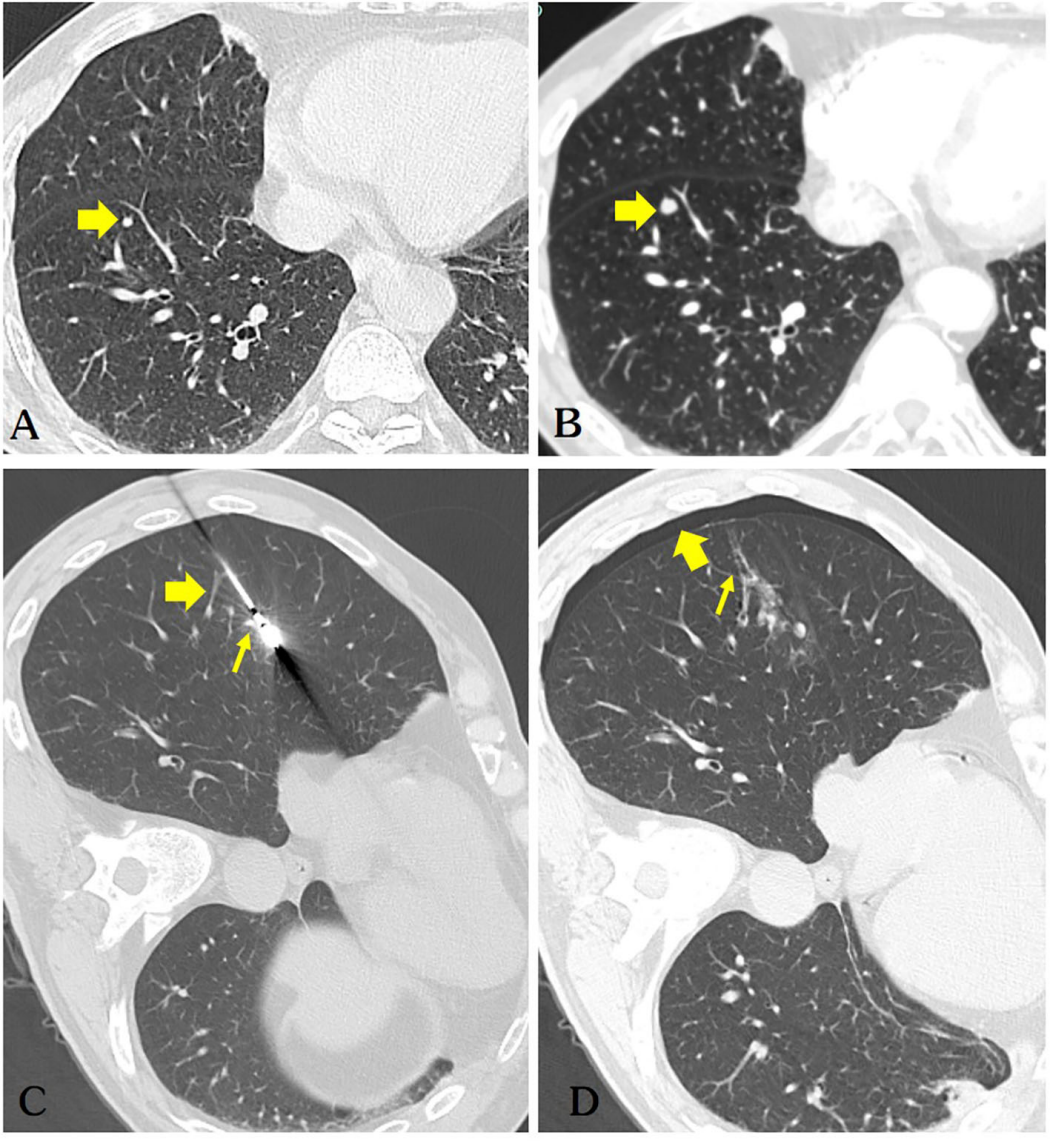
**(A)** The patient’s chest CT scan shows a solid nodule (diameter of 3.1mm) in the right lower lobe of the lung(arrow). **(B)** A repeat chest CT scan after 5 months indicates a significant increase in the size of the nodule (diameter of 5.2mm) in the right lower lobe, highly suspicious for pulmonary metastasis (arrow). **(C)** The microwave antenna accurately punctured the lesion. Retrospective analysis considered puncture of the pulmonary vein(thick arrow) and pulmonary artery(thin arrow). **(D)** The patient developed a small right-sided pneumothorax post-operatively (thick arrow), while air was observed in the ablation needle tract (thin arrow), which is a very dangerous sign.

**Figure 2:**
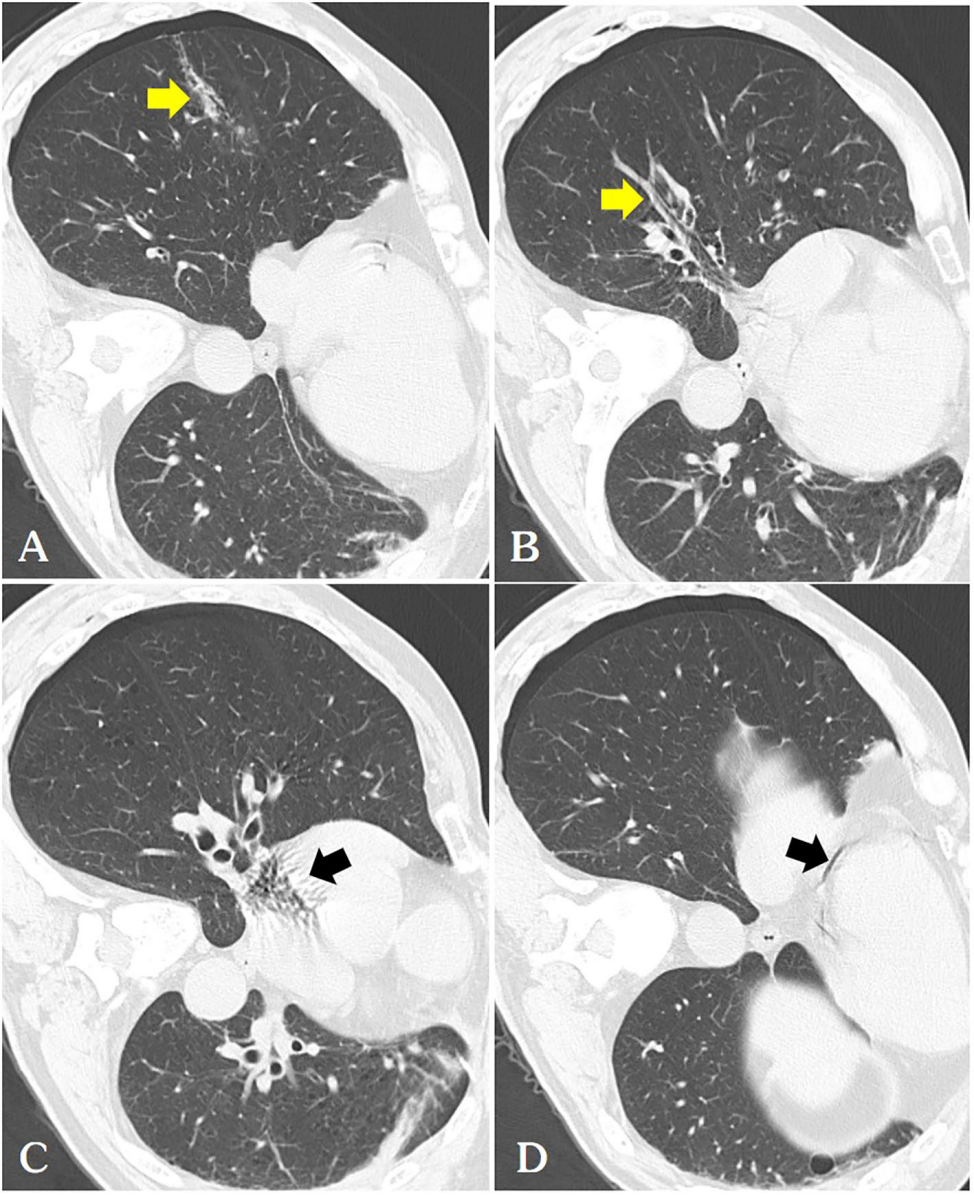
**(A)** We routinely perform a follow-up chest CT immediately after ablation, where we can observe a thicker needle tract(arrow) connecting the pneumothorax with the pulmonary vein [(**B**, arrow)] and pulmonary artery, hence air is observed in the right atrium [(**C** arrow)] and coronary arteries [(**D** arrow)].

**Figure 3:**
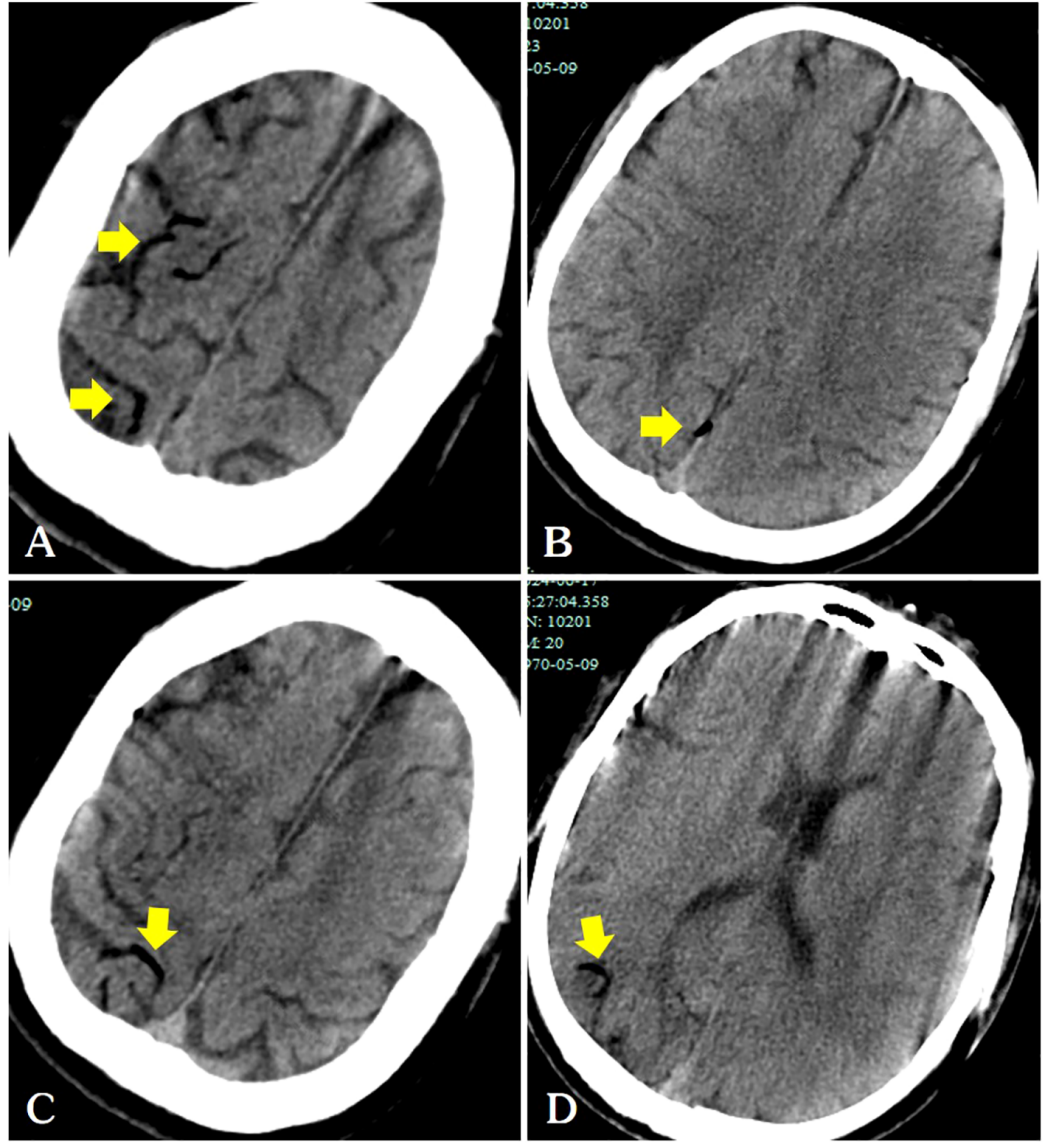
Following the patient’s recovery of breathing and heartbeat, a head CT scan revealed air within the cerebral veins (**A–D**, arrows).

**Figure 4:**
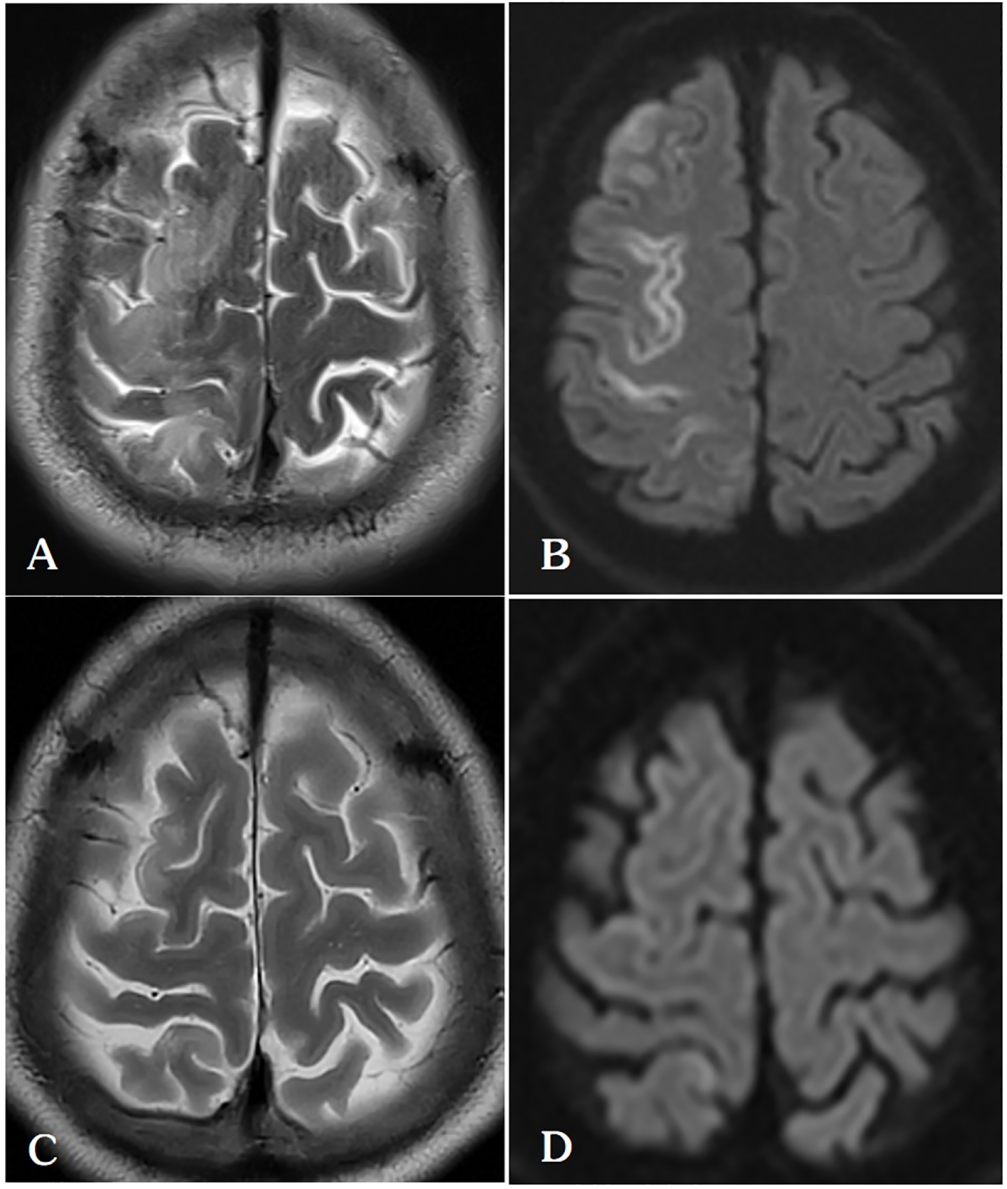
**(A, B)** On the 5th day after lung ablation, the patient’s head MRI indicated venous infarction in the right parietal lobe. **(C, D)** After treatment with anticoagulation, scavenging of oxygen free radicals, and neurotrophic therapy, the patient’s follow-up head MRI on the 30th postoperative day showed a significant reduction in the extent of cerebral infarction in the right frontal lobe.

Our report was supported by informed consent from the patients. A 54-year-old male patient with a solitary metastatic lung lesion originating from rectal cancer in the right lower lobe was referred for computed tomography (CT)-guided MWA. Two years prior, the patient had undergone radical surgery for rectal cancer, followed by adjuvant chemotherapy. Subsequent CT scans revealed the presence of two masses: a 6.3 mm metastatic lesionin the right lower lung lobe and a 10 mm metastatic lesion in the left upper lung lobe. Following consultation with thoracic surgeons, the patient underwent video-assisted thoracoscopic surgery (VATS) for the left lung metastasis one month prior. Postoperative pathology confirmed the left lung lesion as metastatic adenocarcinoma originating from the rectum. Due to the patient’s limited tolerance for a secondary surgery, the 6.3 mm mass in the right lower lobe was scheduled for microwave ablation (MWA) (**Figures 1A, B**). Before the ablation procedure, the patient was prepared for monitoring of heart rate, electrocardiographic data, arterial oxygen saturation, and blood pressure. They were then positioned in a left lateral decubitus position. The entire ablation procedure was performed with the assistance of CT guidance. After local anesthesia of the subcutaneous tissue was achieved with subcutaneous administration of 2% lidocaine, CT-guided MWA was performed using a METI-IVB MWA system (Fuzhong Medical Instrument Co., Ltd. Nanjing, China) and a 16-gauge cooled-shaft antenna, during a breath hold Following penetration of the pleura by the antenna, it was subsequently advanced toward the tumor. To confirm the correct positioning of the ablation antenna, a follow-up CT scan was performed after the needle insertion (**Figure 1C**). Continuous ablation was carried out using a 40W power source for six minutes, aiming to createan ablation zone that was 5–10 mm larger than the tumor site (**Figure 1D**). After the antenna was removed and the patient was assisted into a sitting position, he became agitated and lost consciousness. A CT scan revealed a small volume in the pulmonary vein (**Figure 2B**) and a significant amount of air in the right atrium (**Figure 2C**), as well as air observed in the coronary arteries (**Figure 2D**). Subsequently, the patient experienced cardiac arrest, which was successfully treated with cardiopulmonary resuscitation. Upon regaining consciousness, the patient exhibited complete left hemiplegia and aphasia. A brain CT scan revealed the presence of intracranial air, predominantly located in the right frontal and parietal lobes, indicating a systemic air embolism (**Figures 3A–D**). The chest CT scan showed pulmonary hemorrhage near the puncture needle, along with a minor pneumothorax, estimated to be approximately 10%. The occurrence of a systemic air embolism was confirmed. A subsequent cerebral angiography showed no significant abnormalities. Following this, the patient was transferred to the Department of Neurology for further treatment. Neurologicale examination indicated that the patient was fully conscious. Muscle strength in the left upper extremity was graded as 0, while strength in the left lower extremity was graded as 4. The electrocardiogram (ECG) did not reveal any discernible abnormalities, and laboratory markers related to myocardial infarction, such as nT-BNP, Myocardial enzymes and troponin, were within normal limits. However, the head magnetic resonance imaging (MRI) showed a gyrus-like high signal in the right parietal lobe cortex (**Figures 4A, B**), which could fully explain the neurological symptoms and findings from the physical examination.

In the neurology ward, the patient was administered oxygen at a rate of 6 L/min via a nasal catheter, which is the standard treatment for pneumothorax. His SpO2 remained stable, exceeding 95%. He received Aspirin (antiplatelet), Edaravone (to protect neuronal cells), and Butylphthalide (improving the cerebral blood circulation), along with bedside rehabilitation. Our hospital does not have the necessary infrastructure for hyperbaric oxygen therapy, and the patient’s family declined our recommendation to seek this treatment at an alternative facility. Over time, the muscle strength in patient’s left limb gradually returned to normal, and follow-up head MRI conducted about four weeks later showed a significant reduction in the extent of cerebral infarction compared to previous scans (**Figures 4C, D**). The patient was discharged 33 days after the event, exhibiting no neurological deficits (**Table 1**).

**Table 1:** Timeline of important events.

Time	Event
14:15	Started the ablation
14:30	Chest CT shown air in the right atrium and pulmonary veins, as well as air demonstrated in the coronary arteries
14:32	Loss of consciousness, sudden cardiac and respiratory arrest; CRP
14:30	Respiratory and heartbeat recovery; Supine position and 6 L/min oxygen inhalation
14:35	Head CT shown intracranial air
14:50	Consciousness; Left upper limbs muscle strength was grade 0, and his left lower limbs muscle strength was grade 4.
17:00	NO abnormalities on cerebrovascular DSA
17:35	Transferred to the Department of Neurology
Day 2	Epilepsy; Continuous administration of Debakin
Day 5	Cerebral venous infarction shown on head MRI
Day 13	Transferred to the rehabilitation
Day 29	The scope of cerebral infarction had significantly narrowed on head MRI
Day 33	Discharged without permanent sequelae

CRP, cardiopulmonary resuscitation; DSA, Digital silhouette angiography.

## Discussion

Systemic air embolism is a rare but potentially serious complication that can occur during CT-guided percutaneous microwave ablation of lung lesions. In our experience treating 215 patients with this technology, this is the first case of gas embolism that we have encountered. Similar cases of air embolism following radiofrequency ablated of lung lesions have been reported in the literature. Massive embolism in the left side of the heart described in a previous case report is not always symptomatic (1). Tomohisa et al. (2) described an air embolism during radiofrequency ablation of a pulmonary lesion; fortunately, a brain CT scan shows no evidence of intracranial air attenuation. Aude et al. (3) reported a case of air embolism presenting with myocardial infarction and severe impairment of cardiac function as the initial symptom.

Various mechanisms may explain the entry of air into the coronary artery during surgery. The device uses only a microwaveable antenna, and air in the pleural cavity indirectly enters the pulmonary vein through the puncture needle track. Additionally, intra-alveolar or intra-bronchial air may enter the pulmonary venous circulation. A third potential mechanism is the entry of air into the pulmonary arterial circulation, which subsequently traverses the pulmonary microvasculature to reach the pulmonary venous circulation, even in the absence of an arteriovenous malformation. Our sole explanation for the presence of air in the pulmonary veins is the puncture tract connected the pneumothorax with the pulmonary veins, allowing air to transit between the two (**Figure 2A**). The pulmonary veins expel air directly into the left atrium and left ventricle, subsequently entering the body circulation and coronary arteries.

In our case, air was observed in the surface veins of the cerebral sulcus and sagittal sinus on the patient’s head CT, and a diagnosis of venous cerebral infarction was confirmed on head MRI a few days later. A detailed preoperative examination ruled out a left-to-right cardiac shunt. Air in the pleural cavity may have indirectly enters the pulmonary arteries through the puncture needle track. The mechanism of retrograde cerebral venous air embolism was further supported by the presence of air in the right atrium on chest CT images (**Figure 2C**). The occurrence of venous cerebral air emboli may be attributed to the retrograde movement of air into the jugular veins, particularly in instances where the patient is in an upright position. Intravenous gas may travel through the veins in a direction opposite to the normal blood flow and reach the intracranial venous sinuses if the volume of the air embolus exceeds the capacity of the pulmonary filter (4).

A patient with an air embolism should be immediately placed in the left lateral decubitus position and the Trendelenburg position (5, 6). Some controversy exists regarding the use of the Trendelenburg position for patients with air embolism (7). However a systematic review indicated that patients placed in the Trendelenburg position for air embolism tended to have more favorable outcomes (8). The management of an acute air embolism is dependent on the clinical condition of the patient. In most patients, treatment is supportive and includes high flow oxygen, volume resuscitation, vasopressors, and advanced cardiac life support (ACLS). Hyperbaric oxygen therapy plays a critical role in the successful resuscitation of these patients (5) and has been shown to be an effective treatment for systemic air embolism (9). In our case, hyperbaric oxygen was not used due to the lack of a hyperbaric chamber in our hospital.

In the event that patients present with symptoms indicative of cerebral vascular embolism, such as limb weakness, hemianopia, dysphasia, loss of consciousness, or coronary artery embolism, including acute coronary syndrome, cardiac arrest, shock, and so forth, the potential for air embolism must be considered. Our case highlights the importance of recognizing systemic air embolism and ensuring that facilities are equipped to provide emergency treatment at all times. Although the patient and his family in this case were understanding of the complication, it remains essential to fully informed patients about the severity and unpredictability of air embolism before the ablation procedure. The patient should undergo a chest enhanced CT scan before microwave ablation of the pulmonary lesions. When planning the puncture path, the interventional physician should pay attention to the following two points: first, avoid passing through large pulmonary vessels, especially the pulmonary veins; second, steer clear of cavitary lung lesions. Most of our surgeries are performed under local anesthesia, and the doctor should remind the patient not to cough forcefully. We always perform lung microwave ablation under local anesthesia in the radiology department away from the operating room. This serious complication serves to reinforce the necessity of training experienced anesthesiologists and ensuring that anesthetic units are equipped with adequate supplies for cardiopulmonary resuscitation.

## Conclusion

In conclusion, this case study highlights a potentially severe complication that may occur during lung microwave ablation. It is essential for physicians performing lung biopsies and ablation procedures to be aware of the possibility of unforeseen complications and to have access to emergency guideline for their management.

## Data Availability

The original contributions presented in the study are included in the article/supplementary material. Further inquiries can be directed to the corresponding author.
